# Antimicrobial Peptides for Gram-Negative Sepsis: A Case for the Polymyxins

**DOI:** 10.3389/fimmu.2012.00252

**Published:** 2012-08-15

**Authors:** Sunil A. David

**Affiliations:** ^1^Department of Medicinal Chemistry, University of KansasLawrence, KS, USA

**Figure 1:**
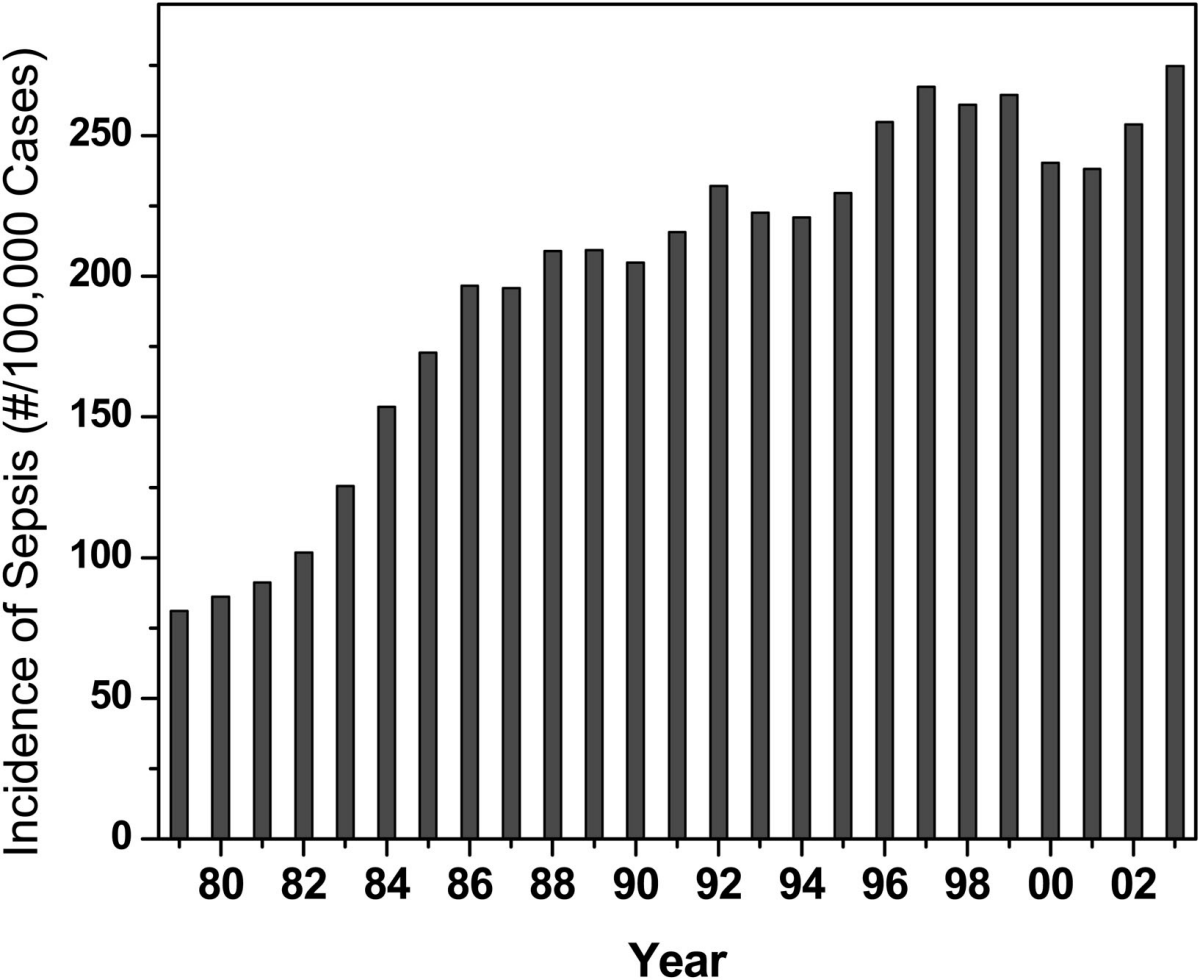
**Incidence of sepsis in the US Data provided by Greg Martin**.

Sepsis, or “blood poisoning” in lay terminology, is a common and serious clinical problem. While fewer than 100 cases were reported prior to 1920 (Felty and Keefer, 1924), it is now the 13th leading cause of overall mortality (Gelfand and Shapiro, 1993) and the number one cause of deaths in the intensive care unit accounting for some 200,000 fatalities in the US annually. The incidence continues to rise in the US (Martin et al., 2003; Figure 1) and worldwide (Moss and Martin, 2004), perhaps due to increased invasive procedures, immunosuppression, and cytotoxic chemotherapy. Mortality associated with sepsis, unfortunately, has essentially remained unchanged at about 45% (Cross and Opal, 1994), despite tremendous strides in antimicrobial chemotherapy, pointing to the absence of therapeutic strategies aimed specifically at the pathophysiology of sepsis. The pathophysiology of the disease is characterized by a systemic inflammatory response syndrome (SIRS), culminating in its frequently fatal sequel, multiple organ dysfunction syndrome (MODS). The systemic inflammatory response is a consequence of dysregulated activation of innate immune effector mechanisms (Castellheim et al., 2009). Counterregulatory mechanisms that are subsequently deployed to dampen the initial overexuberant systemic inflammatory responses are also thought to contribute to the pathophysiology due to late-stage immunosuppressive (hypoinflammatory) phenomena, which render the host unable to eradicate the offending pathogen (Hotchkiss and Karl, 2003; Hotchkiss et al., 2009).

The primary trigger of SIRS in the Gram-negative septic shock syndrome is thought to be endotoxin, a constituent of the outer membrane of all Gram-negative bacteria. Endotoxins consist of a polysaccharide portion and a lipid called lipid A, and are therefore also called lipopolysaccharides (LPS). The polysaccharide portion consists of an O-antigen-specific polymer of repeating oligosaccharide units, the composition of which is highly varied among Gram-negative bacteria. A relatively well-conserved core hetero-oligosaccharide covalently bridges the O-antigen-specific chain with lipid A (Rietschel et al., 1994). Total synthesis of the structurally highly conserved lipid A has been shown to be the active moiety of LPS (Rietschel et al., 1987).

Whereas LPS itself is chemically inert, the presence of LPS in blood (endotoxemia), often a consequence of antibiotic therapy of preexisting bacterial infections (Holzheimer, 2001), is recognized by Toll-like receptor 4 (TLR4; Beutler and Poltorak, 2001; Palsson-McDermott and O’Neill, 2004; Hennessy et al., 2010), a member of a large super-family of pattern recognition receptors (Jounai et al., 2012; Newton and Dixit, 2012; Olive, 2012). Endotoxemia and its sequelae may arise even in the absence of Gram-negative bacterial infections, conditions such as trauma (Saadia et al., 1990), burns (Jones II et al., 1991), and splanchnic ischemia during cardiac surgery (Rocke et al., 1987) increase intestinal permeability, resulting in the spill-over into the portal circulation of LPS from the colon which is abundantly colonized by Gram-negative bacteria. The sensing of LPS, “read by our tissues as the very worst of bad news” (Thomas, 1975), results in a cascade of exaggerated host responses, manifesting in the clinical syndrome characterized by endothelial damage, coagulopathy, loss of vascular tone, myocardial dysfunction, tissue hypoperfusion, and multiple-system organ failure (Balk and Bone, 1989; Bone et al., 1992; Bone, 1993). LPS activates almost every component of the cellular and humoral (plasma protein) limbs of the immune system, resulting in the production of a plethora of proinflammatory mediators, important among which are not only early-phase cytokines such as tumor necrosis factor-α (TNF-α), interleukin-1β (IL-1β), and IL-6 (Dinarello, 1991, 1996) but also late-phase endogenous mediators such as high mobility group box 1 protein (HMGB1; (Wang et al., 1999; Andersson and Tracey, 2011). These cytokines and other mediators act in concert, amplifying the resultant generalized inflammatory processes.

Our understanding of basic mechanisms underlying the cellular response to LPS has increased vastly in recent years. These advances will likely offer novel therapeutic possibilities in the future. However, after more than two decades of intensive effort at evaluating more than 30 investigational compounds, specific therapeutic options for sepsis have remained elusive. Drotrecogin alfa (Xigris^™^, recombinant human activated protein C), an anticoagulant that ameliorates disseminated intravascular coagulation was approved in November 2001 by the FDA, but recently withdrawn due to lack of efficacy (Ranieri et al., 2012; Wenzel and Edmond, 2012). Clinical trials aimed at blocking various proinflammatory mediators including TNF-α, IL-1β, platelet-activating factor, and prostaglandins produced by the activated cellular components have all been disappointing (Zeni et al., 1997), suggesting that targeting downstream cellular inflammatory processes once immune activation has already progressed is unlikely to be of benefit.

It follows, therefore, that the paradigm of proximal, upstream intervention using molecules that specifically block the recognition of LPS by TLR4 would offer attractive therapeutic targets. As mentioned earlier, the polysaccharide portion of LPS is highly variable and serologically distinct for each strain of the same species of Gram-negative organisms. Although anti-O-polysaccharide antibodies afford protection in experimental models where animals are challenged with homologous bacteria (Kim et al., 1988; Siegel, 1995), these are not likely to be of significant clinical value since sepsis runs an acute course before the pathogen is identified and appropriate specific immunotherapy is instituted. The biologically active part of LPS, lipid A, as well as the core oligosaccharide portion are structurally highly conserved across Gram-negative genera, and thus are attractive targets for sequestration, and elimination of circulating LPS would, in principle, prevent the activation of inflammatory cascades (Ziegler et al., 1982; Ziegler, 1988; Ziegler and Smith, 1992). Experimental studies as early as 1968 suggested that antibodies directed toward epitopes in the core region of LPS may be broadly cross-protective against a range of Gram-negative organisms (Chedid et al., 1968). However, neither human (HA-1A; (Ziegler et al., 1991) nor murine (E5; (Bone et al., 1995) anti-lipid A monoclonal antibodies afforded significant protection in large, multiple, placebo-controlled clinical trials (Cross and Opal, 1994). Similarly disappointing results were obtained with core region-directed antibodies (Di Padova et al., 1993; Le Roy et al., 1999). Taken together, these failures could point to intrinsic problems with lipid antigens: poor immunogenicity, inaccessibility of neutralizing epitopes, the generation of non-specific cross-reactive antibodies against irrelevant hydrophobic epitopes (Vaarala et al., 1988), and potential problems with the antibody molecule itself: predominant intravascular compartmentalization, and possible tissue damage induced by activation of complement. Non-immunological blockade of LPS recognition using TLR4 “blockers” is therefore an alternative strategy, a premise that has indeed been explored with molecules structurally related to lipid A, but acting as TLR4-specific antagonists (Christ et al., 1995; Kawata et al., 1999; Wittebole et al., 2010; Ehrentraut et al., 2011; Tidswell and Larosa, 2011), but unfortunately, do not appear promising (Williams, 2012). It is not known whether the lack of efficacy is attributable to its physicochemical properties (high lipophilicity; (Christ et al., 1995) and consequent partitioning into plasma lipoproteins, with loss of activity (Rose et al., 2000).

As mentioned earlier, the structurally invariant and biologically active center of LPS, lipid A, is a logical therapeutic target for neutralization. Lipid A is composed of a hydrophilic, negatively charged *bis*-phosphorylated di-glucosamine backbone, and a hydrophobic domain of six (*E. coli)* or seven (*Salmonella*) acyl chains. The anionic amphiphilic nature of lipid A enables it to interact with a variety of cationic hydrophobic ligands (Vaara and Vaara, 1983; Peterson et al., 1985; Rocque et al., 1988). Polymyxin B (PMB), a cationic amphiphilic cyclic decapeptide antibiotic isolated from *Bacillus polymyxa* (Storm and Rosenthal, 1977), has long been recognized to bind lipid A (Morrison and Jacobs, 1976), and neutralize its toxicity in animal models of endotoxemia (Stokes et al., 1989; Durando et al., 1994; Yao et al., 1995). Although PMB is a commonly used topical antibiotic, it is nephro- and oto-toxic, which, while hitherto precluding its use as an LPS-neutralizer in patients with sepsis, has stimulated the search for non-toxic PMB analogs (Rustici et al., 1993; Porro et al., 1998), PMB derivatives (Vaara, 1983; Viljanen et al., 1991), as well as other structurally diverse cationic amphiphilic peptides (Rustici et al., 1993; Porro, 1994; Iwagaki et al., 2000; Scott et al., 2000; Jerala and Porro, 2004) as candidate LPS-binding agents. Notably, a hemoperfusion cartridge based on PMB covalently immobilized via one of its NH_2_ groups to a polystyrene based fiber became available in Japan in late 2000 for clinical use (“Toraymyxin^™^,” Toray Industries Inc., Tokyo; (Nakamura et al., 1999, 2002, 2003). In the EUPHAS randomized clinical trial, PMB hemoperfusion alongside conventional therapy was found to improve organ dysfunction and reduce 28-days mortality in subsets of patients with sepsis arising from intra-abdominal Gram-negative infections (Cruz et al., 2009). Whilst the utility of Toraymyxin provides a clinically validated proof-of-principle for the value of sequestering circulating LPS (Rimmele and Kellum, 2011), opportunities for extracorporeal hemoperfusion may be infrequent due to unfavorable hemodynamic parameters.

Given that the only encouraging lead for the management of sepsis to date appears to be PMB hemofiltration, it is perhaps useful to re-examine PMB itself, as well as its structurally closely related congener, polymyxin E (or colistin). PMB and polymyxin E differ one from the other by a single amino acid (D-Phe in PMB; D-Leu in PME; (Kwa et al., 2007), and are similar in their *in vitro* antimicrobial activity, clinical efficacy, and toxicity (Oliveira et al., 2009). Although banished to a topical-use-only status on account of its systemic toxicity, the polymyxins are rapidly re-emerging as last-resort parenteral antibiotics for the management of infections with extensive drug resistant strains of *Pseudomonas aeruginosa* (Zavascki et al., 2007; Michalopoulos and Falagas, 2008; Giamarellou and Poulakou, 2009), *Acinetobacter baumannii* (Gales et al., 2011; Fitzpatrick et al., 2012), and *Burkholderia* species (Gales et al., 2011). The increased use of these peptide antibiotics has led to a careful re-examination of its purported toxicity (Falagas and Kasiakou, 2006). Evaluation in diverse clinical settings (Ramasubban et al., 2008; Nation and Li, 2009; Lim et al., 2010; Durakovic et al., 2011; Spapen et al., 2011; Cho et al., 2012) indicate that tolerability of the antibiotic is acceptable, and the toxicity is less than previously thought. Of particular interest is a recent report (Mizuyachi et al., 2011) detailing the safety and pharmacokinetic evaluation of intravenous colistin as the methanesulfonate prodrug (Bergen et al., 2006) in healthy human volunteers. Urinary *N*-acetyl-β-D-glucosaminidase (a biomarker for early renal damage) showed only transient and reversible increases at doses eliciting plasma concentrations (free area under the concentration-time curve/MIC) of drug that were predicted to be bactericidal (Mizuyachi et al., 2011). Since the antimicrobial effects of the polymyxins are a direct consequence of their binding to lipopolysaccharide (Morrison and Jacobs, 1976; Nikaido and Vaara, 1985), it is reasonable to assume that the plasma concentration of the drug would be of such magnitude as to be sufficient for sequestering LPS.

Erasmus’ adage, “*Malo nodo, malus quærendus cuneus”* (for a hard knot a hard tool must be sought), rings perhaps particularly true for sepsis for which a single tractable lead toward a validated therapeutic approach is yet to be found, mortality continues to be unacceptably high, and a survey of the research landscape in the field conjures up images of a bleak battlefield strewn with the corpses of many a failed approach. The venerable polymyxins, never subjected to modern regulatory requirements when they were first introduced, and exiled for the last 50 years, are making their way back into the clinic as parenteral antibiotics. Much is being learned as they are re-examined with the rigor and precision of modern methods of pharmacokinetic (Couet et al., 2012) and pharmacodynamic (Mizuyachi et al., 2011) analyses. Perhaps there is a case to be made for a careful risk-benefit evaluation of the polymyxins in Gram-negative sepsis.
